# Silica Precipitation in a Wet–Dry Cycling Hot Spring Simulation Chamber

**DOI:** 10.3390/life10010003

**Published:** 2020-01-14

**Authors:** Andrew Gangidine, Jeff R. Havig, Jeffrey S. Hannon, Andrew D. Czaja

**Affiliations:** 1 Department of Geology, University of Cincinnati, Cincinnati, OH 45221, USA; hannonjs@mail.uc.edu (J.S.H.);; 2 Department of Earth and Environmental Sciences, University of Minnesota, Minneapolis, MN 55455, USA; jhavig@umn.edu

**Keywords:** hydrothermal, origin of life, early earth, hot spring, simulation chamber, silica, precipitation, model

## Abstract

Terrestrial hot springs have emerged as strong contenders for sites that could have facilitated the origin of life. Cycling between wet and dry conditions is a key feature of these systems, which can produce both structural and chemical complexity within protocellular material. Silica precipitation is a common phenomenon in terrestrial hot springs and is closely associated with life in modern systems. Not only does silica preserve evidence of hot spring life, it also can help it survive during life through UV protection, a factor which would be especially relevant on the early Earth. Determining which physical and chemical components of hot springs are the result of life vs. non-life in modern hot spring systems is a difficult task, however, since life is so prevalent in these environments. Using a model hot spring simulation chamber, we demonstrate a simple yet effective way to precipitate silica with or without the presence of life. This system may be valuable in further investigating the plausible role of silica precipitation in ancient terrestrial hot spring environments even before life arose, as well as its potential role in providing protection from the high surface UV conditions which may have been present on early Earth.

## 1. Introduction

In recent years, terrestrial (land-based) hot springs have gained substantial support as environments where life could have arisen on Earth (e.g., [1,2,3]). In particular, the ability for terrestrial hot springs to cycle between wet and dry conditions has been highlighted as a notable advantage over sub-aqueous hydrothermal systems and have been shown to possess the ability to promote or facilitate complex prebiotic chemical reactions including protocell formation, polymerization, etc. [1,2]. As with most prebiotic science, it is often challenging to draw parallels between the ancient, lifeless Earth, and the modern life-abundant Earth. Terrestrial hot springs are no exception, as these environments contain ubiquitous life. Hot spring sources commonly house extremophilic microorganisms, outflow vents are colonized by both phototrophic and chemotrophic organisms, and even siliceous hot spring deposits are home to endolithic microbial communities. These environments are inseparable from chemical traces of life, making it difficult to tease apart non-biologic components from biologically associated components.

**Figure 1:**
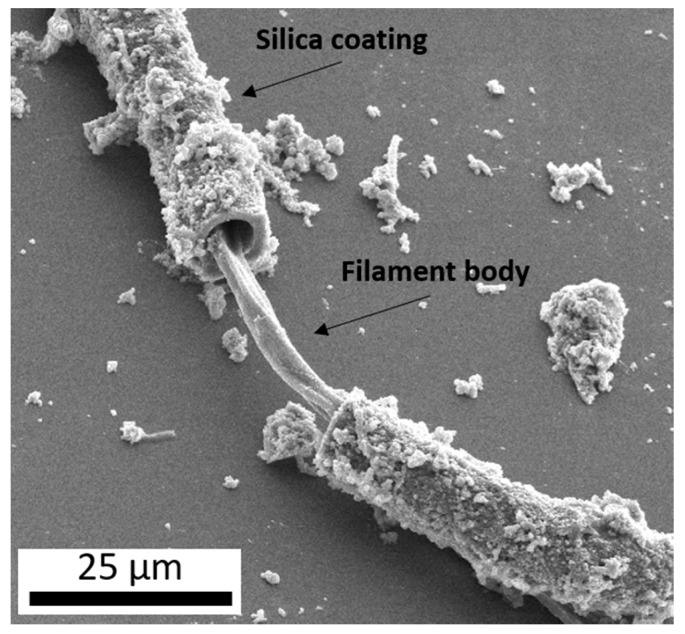
A secondary electron image (10 kV) of a microbial filament collected from an outflow stream of Steep Cone, a circumneutral hot spring in Yellowstone National Park, displaying a coating of botryoidal silica surrounding the filament body.

In many terrestrial hydrothermal systems, spring water is supersaturated with respect to amorphous silica (SiO_2_) [4]. Silica is also thought to play an important role in synthesizing important prebiotic molecules, specifically when subjected to dehydration and rehydration cycles [5,6,7] which can precipitate out of solution as the water temperature drops with distance from the source. This silica precipitate can entomb and preserve microbial life [8], but can also be observed to be intimately associated with extant microbes (Figure 1) through silica-coatings [9,10,11]. While the exact functions/benefits of these coatings are not completely understood, they may provide some degree of UV protection as silica can shield from intense UV due to a high radiation absorbance [12], a similar strategy employed by many microbial communities in hot spring systems which thrive as endolithic (within-rock) communities boring into the surface of siliceous sinter [13,14], or as hypolithic (under-rock) communities living under millimeters of loose siliceous sinter [15].

**Figure 2:**
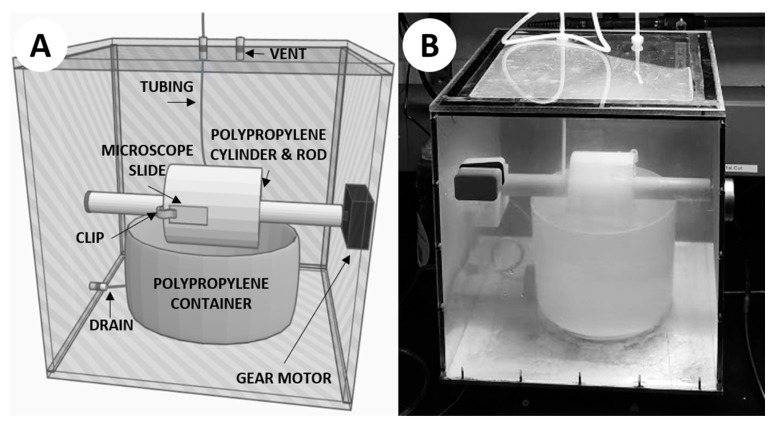
The hot spring simulation chamber. (**A**) 3D rendering of the simulation chamber. (**B**) Photograph of the simulation chamber mid-experiment.

Here we present a relatively simple hot spring “simulation chamber” (Figure 2) which can be used to precipitate silica in geochemical conditions similar to those which could be found in modern hot spring systems (e.g., Table 1). This system precipitates silica through the simple action of microscope slides being attached to a rotating cylinder which is periodically submerged into a carboy of synthesized hot spring fluid and then exposed to air within the chamber. The fluid is gravity fed through autoclavable tubing into the polypropylene container by a carboy above the system. This system can be used to precipitate silica both with and without the addition of microbial organisms, and may prove useful for laboratory-based experiments seeking to investigate questions relating to the potential benefits of silica precipitation in terrestrial hot spring environments.

## 2. Materials and Methods

**Figure A1:**
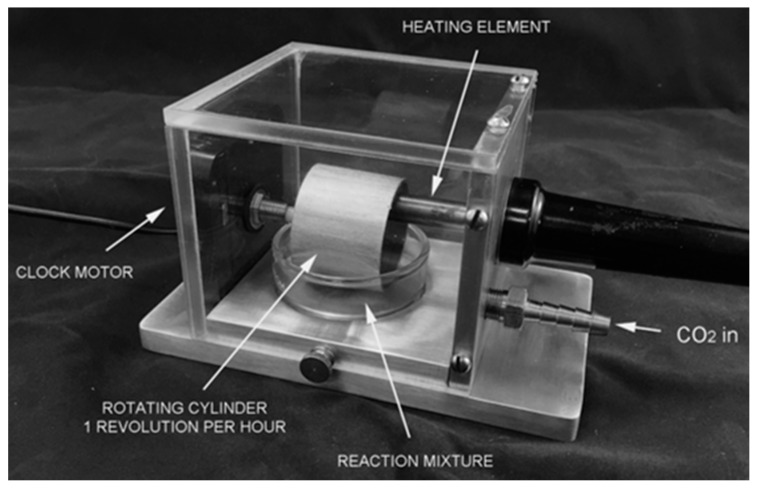
An early wet–dry cycling apparatus prototype developed by Prof. David Deamer to promote monomer polymerization. This apparatus consists of a hollow stainless-steel cylinder, which rotates once per hour via the minute hand of a clock motor. The lower portion of the cylinder rotates into a reaction mixture placed into the dish below. A heating element is used to heat the cylinder to 80°C. CO_2_ or N_2_ enters the apparatus at a low rate (<5 ccs/s) to carry off the water produced by the condensation reactions that polymerize mononucleotides. A typical reaction mixture holds 10 mM mononucleotides in their acid form, together with either 10 mM phospholipid or 100 mM ammonium chloride, both of which promote polymerization. As the cylinder rotates out of the reaction mixture, a thin film of the mixture is picked up on the burnished surface of the cylinder and would be quickly dried by the heat so that the monomers could polymerize. The dried film then re-dissolves when the cylinder rotates back into the mixture. To prevent the entire 15 mL from evaporating, a perfusion pump delivers water at the same rate it evaporated.

The construction of this simulation chamber was based on a prototype design conceived by Prof. David Deamer of UC Santa Cruz to facilitate the polymerization of monomers via wet–dry cycling (Figure A1 in Appendix A). The newly constructed apparatus we used for this study was designed to simulate a modern hot spring more closely, particularly an outflow stream where life is often observed and silica precipitation is commonplace. The chamber used the same rotating-cylinder technique, with some notable differences (Figure 2). A 12 × 12 × 0.25 inch aluminum sheet served as the base of the chamber, in order to efficiently transfer heat from a hot plate directly beneath the chamber. The walls of the chamber were constructed from 12 × 12 × 0.25 inch polycarbonate sheets affixed to the aluminum base, with the top cover being affixed with additional gasket material for sealing purposes. The cylinder was machined from polypropylene, with a 1-inch bore through the center in order to be mounted on a 1-inch diameter rotating rod. The rod was attached to a 1 RPM gear motor affixed through the outside of the chamber. A polypropylene container was placed directly below the rotating rod, so the cylinder could be partially submerged when the container is filled with fluid. Plastic clips were attached to the cylinder to mount 1 × 3 inch glass microscope slides so that they could be rotated into the fluid within the container and out again as the motor spun the rod. A small vent with 47-mm membrane filter (0.45-µm pore size) was placed on top of the system to relieve pressure while keeping the chamber clean from outside contaminants. Autoclavable tubing was used to deliver the simulated hot spring fluid from a polypropylene carboy mounted above the simulation chamber. The fluid would be added directly into the polypropylene container in the chamber via autoclavable tubing and could be removed from the container using a drain on the bottom side of the polypropylene container which was fed through the side of the chamber via autoclavable tubing. The completed chamber was assembled in a fume hood, and each component of the system was autoclaved to ensure sterilization, acid washed using a 10% solution of trace-metal grade (<1 ppb) nitric acid for ≥3 days, and triple-rinsed using 18.2 MΩ/cm deionized water.

The simulated hot spring fluid used an extra-pure sodium silicate solution to serve as a silica source. The solution was buffered with sodium bicarbonate and used sodium hydroxide and hydrochloric acid in order to achieve a circumneutral pH. The parameters set for the simulated hot spring fluid used in these experiments (Table 1) were based off geochemical data obtained from Steep Cone, a circumneutral hot spring in Yellowstone National Park. It should be noted, however, that hot springs exhibit a wide range of geochemical conditions (e.g., pH, silica content, other dissolved species, temperature, conductivity; see [16]), so these conditions are by no means exhaustive. 

Two experimental conditions were used to precipitate silica from the simulated hot spring fluid. One involved only silica precipitation under sterile conditions, and the other involved precipitation in the presence of added microbes (a mix of cyanobacteria, *Gleocapsa*, *Oscillatoria*, *Anabaena*, *Fischerella*, and *Gleotrichia*) which were streaked onto the 1 × 3 inch glass microscope slides before being clipped to the rotating cylinder. In the second experiment, these microbes were chosen for being metabolically similar to phototrophic organisms commonly found in hot spring outflow vents. For both experiments, the simulated hot spring fluid was heated to 50 °C, a common temperature of phototrophic microbial community colonized outflow streams in terrestrial hot springs.

Scanning electron microscopy (SEM) was performed using a SCIOS Dual-Beam Scanning Electron Microscope in the Advanced Materials Characterization Center at the University of Cincinnati. Both secondary electron and Everhart-Thornley Detector (i.e., “ET-detector”, a combination of secondary electron and back-scattered electron detector) imaging was performed under high vacuum using Pt/Au coating and voltages ranging from 5 to 10 kV.

## 3. Results

### 3.1. Abiotic Experiment

**Figure 3:**
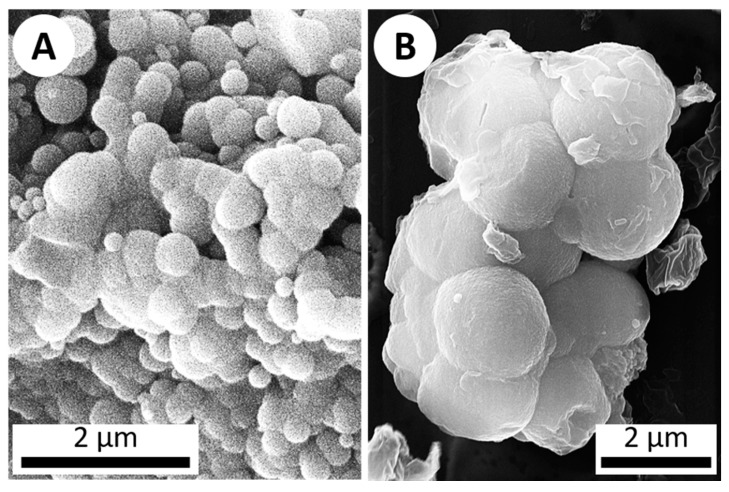
SEM imagery of botryoidal silica structures. (**A**) A secondary electron image (10 kV) of botryoidal silica precipitate from a sinter sample collected from Steep Cone hot spring in Yellowstone National Park. (**B**) ET-detector image (20 kV) of silica which precipitated on a microscope slide after 1 week in the hot spring simulation chamber (abiotic), displaying the same botryoidal texture characteristic of silica precipitation.

The first objective was to experimentally precipitate silica using the hot spring simulation chamber. Since silica commonly interacts with life (e.g., via precipitation/deposition, coating organisms, etc.) in hydrothermal settings, while some silica precipitation may be biologically-influenced (e.g., via metabolic re-precipitation), it is mostly thought to occur via abiologic processes such as cooling and dehydration [17]. Silica can precipitate via dehydration, but the relatively fast rotation (1 RPM) combined with a temperature of 50 °C and minimal venting meant the system would remain humid. Despite this, after seven days, micron-scale silica precipitates were observed, with the same characteristic botryoidal texture found in silica precipitates from modern hot spring environments (Figure 3) [9,10,11,16].

### 3.2. Added Microbial Life Experiment

**Figure 4:**
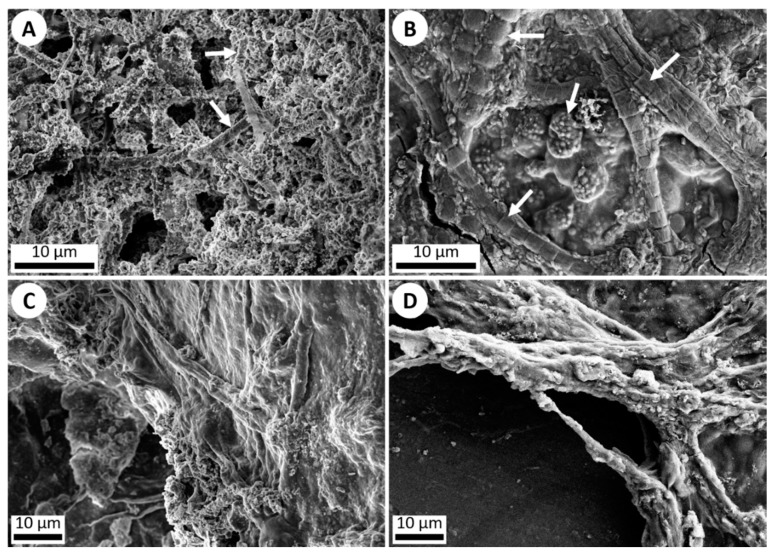
SEM imagery of silica precipitation and microbial life. (**A**) ET-detector image (5 kV) of silica precipitation exhibited around individual microbes (white arrows) in a Yellowstone National Park hot spring outflow stream. (**B**) ET-detector image (5 kV) of silica precipitation exhibited around various microbes (white arrows) which were added onto a microscope slide and run through the hot spring simulation chamber for 1 week. Silica precipitation can be observed starting to cover the organisms. (**C**) ET-detector image (5 kV) of a dense microbial mat collected from an outflow stream in Yellowstone National Park, showing silica precipitation. (**D**) ET-detector image (10 kV) of a dense microbial mat of various non-hot spring cyanobacteria added onto a microscope slide and run through the hot spring simulation chamber for 1 week, once again exhibiting silica precipitation coating the mat.

**Figure 5:**
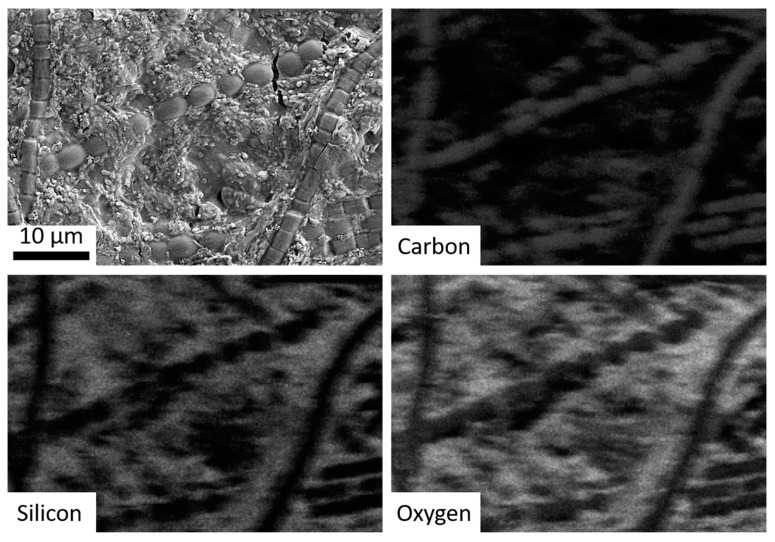
SEM imagery of bacteria run through the hot spring simulation chamber, with subsequent panels showing EDS mapping for carbon (co-localized with the bacteria), silicon, and oxygen (i.e., silica).

The second objective was to simulate the coating of microbes with precipitated silica in hot springs. This was accomplished by the addition of various cyanobacteria to the hot spring simulation chamber. A cyanobacteria mixture of Gleocapsa, Oscillatoria, Anabaena, Fischerella, and Gleotrichia was streaked onto microscope slides attached to the rotating cylinder. After seven days, the cyanobacteria were imaged via SEM and Energy-dispersive X-ray spectroscopy (EDS) which showed that the bacteria were covered with/surrounded by botryoidal silica precipitate similar to that observed in microbial samples obtained from hot spring outflow streams in Yellowstone National Park (Figure 4 and Figure 5) [10,11,16].

## 4. Discussion

### 4.1. Silica and Its Possible Role in the Origin of Life

This wet–dry cycling simulation chamber can be used to produce abiotic silica precipitation without the need for total dehydration in relatively short periods of time, and thus more accurately represents “natural” conditions that would be conducive to life. Thus, this system can even be used to study live organisms in a recreated hot spring setting, which could realistically survive throughout the course of an experiment. This allows for a unique, easy to assemble simulation chamber to produce and study silica precipitation with or without the addition life. When organisms are added to the system, it can successfully be used to at least partially replicate silica-coating observed in hot spring systems.

The development of life on early Earth would likely have been a scenario fraught with obstacles. Natural conditions that may have aided in this process may play a vital role in the formation and subsequent colonization of life on Earth. UV conditions on early Earth are thought to be a key boundary for abiogenesis, and are largely dependent on various factors including atmospheric absorption, attenuation by water, and stellar variability [18,19]. The exact UV conditions throughout early Earth are still debated, and it is not yet known whether or not surface UV was hostile to life, or whether life may have exhibited some kind of defense against harmful UV, or instead dwelled in non-surface environments which would have been more shielded from UV [20,21]. If UV conditions on the early Earth were indeed harmful to life, silica precipitated from hydrothermal environments could have provided some protection from harmful UV while allowing the benefits of surface life [15]. By being able to precipitate silica through wet–dry cycling (hypothesized as being crucial for increasing complexity in prebiotic chemistry [1]), the simulation chamber we present here may be valuable in testing hypotheses regarding the ability of surface hot springs to shield from harmful UV radiation [15].

### 4.2. Wet–Dry Cycling Simulations

Understanding the transition of the Earth from a lifeless to a life-filled world has been a question at the forefront of science, notably address in Charles Darwin’s letter to his friend J. D. Hooker in 1871, where he supposed a “warm little pond” where life may have originated [22]. In recent years, Darwin’s “warm little pond” hypothesis for the origin of life has gained support, with researchers attempting to recreate such conditions in the lab. To this day, laboratory-based simulation chambers attempt to simulate the prebiotic Earth. Early experimentation showed the value of wet–dry cycling, whereupon liposomes subjected to wet–dry cycling resulted in the formation of multilamellar structures trapping solute during drying phases, and the lamellae-forming vesicles containing solute during rehydration [23]. Recent advances have built upon this concept, showing that molecular systems subjected to hydration and dehydration cycles in prebiotic analogue systems undergo chemical evolution (e.g., polymerization), encapsulation, and combinatorial selection [1]. These results have been replicated by other laboratories equipped with instrumentation which also supports wet–dry cycling, such as a new “planet simulator” with a similar “primordial soup” added into it, to simulate seasons in a volcanic surface environment (i.e., hot during the day yet cold at night). The simulator eventually showed the formation of cell-like structures which incorporated genetic material, similar to what was observed in prior wet–dry experiments [24]. Other laboratory-based wet–dry cycling experiments have likewise been used to suggest that wet–dry cycling could be used to form polypeptides (key components of modern life) on the prebiotic Earth [25]. Through such laboratory-based wet–dry cycling experimental work, terrestrial hot springs have emerged as convincing environments which may have harbored the origin of life on Earth and will undoubtedly continue to be investigated. We propose our new wet–dry cycling chamber as an additional tool to test the validity of hypotheses involving hydrothermal systems and wet–dry cycling, specifically those which propose an important role for silica precipitation. 

### 4.3. Variation and Future Studies

Hot spring systems are complex and highly variable [16]. Even the same spring can vary wildly in terms of geochemistry from one year to the next [26,27]. Several variations of this system could be attempted in the future to test more specific hypotheses such as running the system under anoxic atmospheric conditions which may more closely resemble conditions of the ancient Earth, testing a wider range of geochemical conditions (e.g., more acidic, more alkaline, different element concentrations), testing various temperatures, and adding other microorganisms. By testing such variables, we may better understand terrestrial hot spring systems and the vast roles they have been hypothesized to play in potentially facilitating the origin, evolution, and dispersal of life on the early Earth (e.g., [1,12]).

## Figures and Tables

**Table 1:** Simulated hot spring geochemistry.

Parameter	Value
pH	7.67
Temperature	50 °C
Conductivity	382 μS/cm
Salinity	0.19 ppt
Total Dissolved Solids	~270 ppm
Silica	~250–290 ppm
